# Obituary

**Published:** 1850-09

**Authors:** 


### ARTICLE VII.

### OBITUARY.

Died of Peritonitis, in Sacramento City, Cal., on the 14th of July last, JOSIAH B. HERRICK: M. D., late Demonstrator of Anatomy in Rush Medical College, in the 29th year of his age.

Dr. Herrick was a young man of much promise. Endowed with a quick and philossphical mind, a strong constitution, and with great energy and zeal in the prosecution of his designs, he had already a high reputation in the profession. His affability and kindness had secured to him a large circle of friends, who wlll mourn for his untimely fate.

—On the 14th of August, in Chicago, G. W. WENTWORTH, M. D.

—Died recently, at Cincinnati, O., JOHN T. SHOTWELL, M. D., Professor of Anatomy in the Medical College of Ohio.

